# Transfusion practice in anemic, non-bleeding patients: Cross-sectional survey of physicians working in general internal medicine teaching hospitals in Switzerland

**DOI:** 10.1371/journal.pone.0191752

**Published:** 2018-01-30

**Authors:** Michelle von Babo, Corinne Chmiel, Simon Andreas Müggler, Julia Rakusa, Caroline Schuppli, Philipp Meier, Manuel Fischler, Martin Urner

**Affiliations:** 1 Department of Internal Medicine, Waid City Hospital, Zurich, Switzerland; 2 Institute of Primary Care, University of Zurich, Zurich, Switzerland; 3 Anthropological Institute and Museum, University of Zurich, Zurich, Switzerland; 4 Applied Aquatic Ecology, Swiss Federal Institute of Environmental Science and Technology (EAWAG), Dübendorf, Switzerland; 5 Interdepartmental Division of Critical Care Medicine, University of Toronto, Toronto, Canada; University of Florida, UNITED STATES

## Abstract

**Background:**

Transfusion practice might significantly influence patient morbidity and mortality. Between European countries, transfusion practice of red blood cells (RBC) greatly differs. Only sparse data are available on transfusion practice of general internal medicine physicians in Switzerland.

**Methods:**

In this cross-sectional survey, physicians working in general medicine teaching hospitals in Switzerland were investigated regarding their self-reported transfusion practice in anemic patients without acute bleeding. The definition of anemia, transfusion triggers, knowledge on RBC transfusion, and implementation of guidelines were assessed.

**Results:**

560 physicians of 71 hospitals (64%) responded to the survey. Anemia was defined at very diverging hemoglobin values (by 38% at a hemoglobin <130 g/L for men and by 57% at <120 g/L in non-pregnant women). 62% and 43% respectively, did not define anemia in men and in women according to the World Health Organization. Fifty percent reported not to transfuse RBC according to international guidelines. Following factors were indicated to influence the decision to transfuse: educational background of the physicians, geographical region of employment, severity of anemia, and presence of known coronary artery disease. 60% indicated that their knowledge on Transfusion-related Acute Lung Injury (TRALI) did not influence transfusion practice. 50% of physicians stated that no local transfusion guidelines exist and 84% supported the development of national recommendations on transfusion in non-acutely bleeding, anemic patients.

**Conclusion:**

This study highlights the lack of adherence to current transfusion guidelines in Switzerland. Identifying and subsequently correcting this deficit in knowledge translation may have a significant impact on patient care.

## Introduction

Transfusion of red blood cells (RBC) is performed daily in hospitals around the world [1]. Blood transfusions, however, can be associated with multiple disadvantages due to potential adverse events, high costs and limited availability [2, 3]. For many decades, the arbitrary threshold for RBC transfusion was set at a hemoglobin (Hb) level of < 100 g/L and a hematocrit of < 30% [4–6]. The results from the TRICC trial, supporting the restriction of RBC transfusions in specific patient populations, has led to a rethinking [7]. Hence, lower Hb thresholds of 60–80 g/L have been suggested in most patients [7–11]. At present, a broad variety of different transfusion guidelines are available [12–15]. They all emphasize that not only Hb levels, but also patient-related factors (e.g. age, comorbidities, risk of cardiac ischemia) and heterogeneity of anemia (acute versus chronic) should be considered in the decision to transfuse. The amount of transfused RBC differs greatly among European countries [16]. The median transfusion rate in Switzerland is 38.8 units of RBC per 1000 inhabitants, which is slightly above the median transfusion rate of other European countries (36.9 units of RBC per 1000 inhabitants in 2011) [16]. Transfusion practices do not only differ between countries, but also vary highly among individual hospitals in a single country [17]. Only sparse data are available describing the transfusion practices in anemic patients without acute blood loss. In this cross-sectional study, the self-reported practice of physicians working in all general internal medicine teaching hospitals in Switzerland was evaluated, investigating the definition of anemia, thresholds, triggers and effects of RBC transfusion, as well as knowledge and implementation of published or internal transfusion guidelines. We hypothesized that transfusion practice is heterogeneous and differs between regional areas of Switzerland. We also assumed that adherence to current published transfusion guidelines is poor in Switzerland.

## Materials and methods

### Ethical approval

The study protocol has been reviewed by the cantonal research ethics board. The board issued a certificate of non-objection for the study and stated that specific approval was not required according to the Federal Act on Human Research involving Human Beings in Switzerland, since no patients were involved in our study. Participating physicians were anonymized to the study investigators.

### Study participants

A prospective, cross-sectional online-survey was conducted between May 1, 2013, and Sept 30, 2013, in all Swiss hospitals offering acute care, in which resident physicians are trained in the field of general internal medicine according to the regulations of the Swiss Medical Association (FMH) [18]. We excluded hospitals, which were specialized in rehabilitation programs not providing acute care. All 113 eligible hospitals were contacted via an initial letter to the head of the department, followed by a reminder via e-Mail two months later. Both resident and attending physicians were asked to fill out an online questionnaire, which was available in German, French and Italian.

### Questionnaire design

The questionnaire consisted of a 15-item test with ordinal scales for most of the answers. The questionnaire items were designed to cover the current practice of diagnosing and defining anemia (3 items) and treatment of anemia using RBC (6 items). Additional questions addressed the knowledge of published or clinic internal guidelines (3 items). Three vignette questions were created regarding the hemoglobin threshold to administer RBC in different clinical situations (patients without any known risk factors, patients with stable and patients with symptomatic coronary artery disease, and patients with symptomatic anemia). Two external experts in the field were asked to review all items of the questionnaire. A pilot test was conducted in the Waid City Hospital, Zurich, in a subset of ten physicians. The web-based online questionnaire was developed using HTML and CSS mark-up languages for the visual interface. The data was submitted to a MySQL database after a basic plausibility check using the PHP scripting language. A static version of the questionnaires is included in the supplementary information of the manuscript (**S1 Appendix**).

### Statistical analysis

R (R Core Team, 2014) with the lme4 package [19] and the ordinal package [20] were used to perform linear mixed effects or cumulative link mixed model analyses assessing the impact on answers to questionnaire items regarding the diagnosis and treatment of anemia, as well as the three case vignettes (**S1, S2, S3, S4, S5 and S6 Tables**). As fixed effects, we entered gender, clinical experience, clinical position, place of study, type of clinic (university vs. non-university hospital) into the models. The respective cantonal region was introduced in the mixed models using a random intercept. Parameter-specific p-values were obtained as described elsewhere [21–23]. Results were presented as odds ratios with their corresponding 95% intervals. Cumulative link models are also known as proportional odds models [24]: positive coefficients of cumulative link models (corresponding to odds ratios > 1) indicate that a higher predictor value increases the value on the ordinal scale, i.e., rating in higher categories is more likely. The packages ggplot2, maptools, and sp were used to draw the figures [25–27]. A p-value ≤ 0.05 was considered statistically significant.

## Results

### Characteristics of study participants

**Fig 1:**
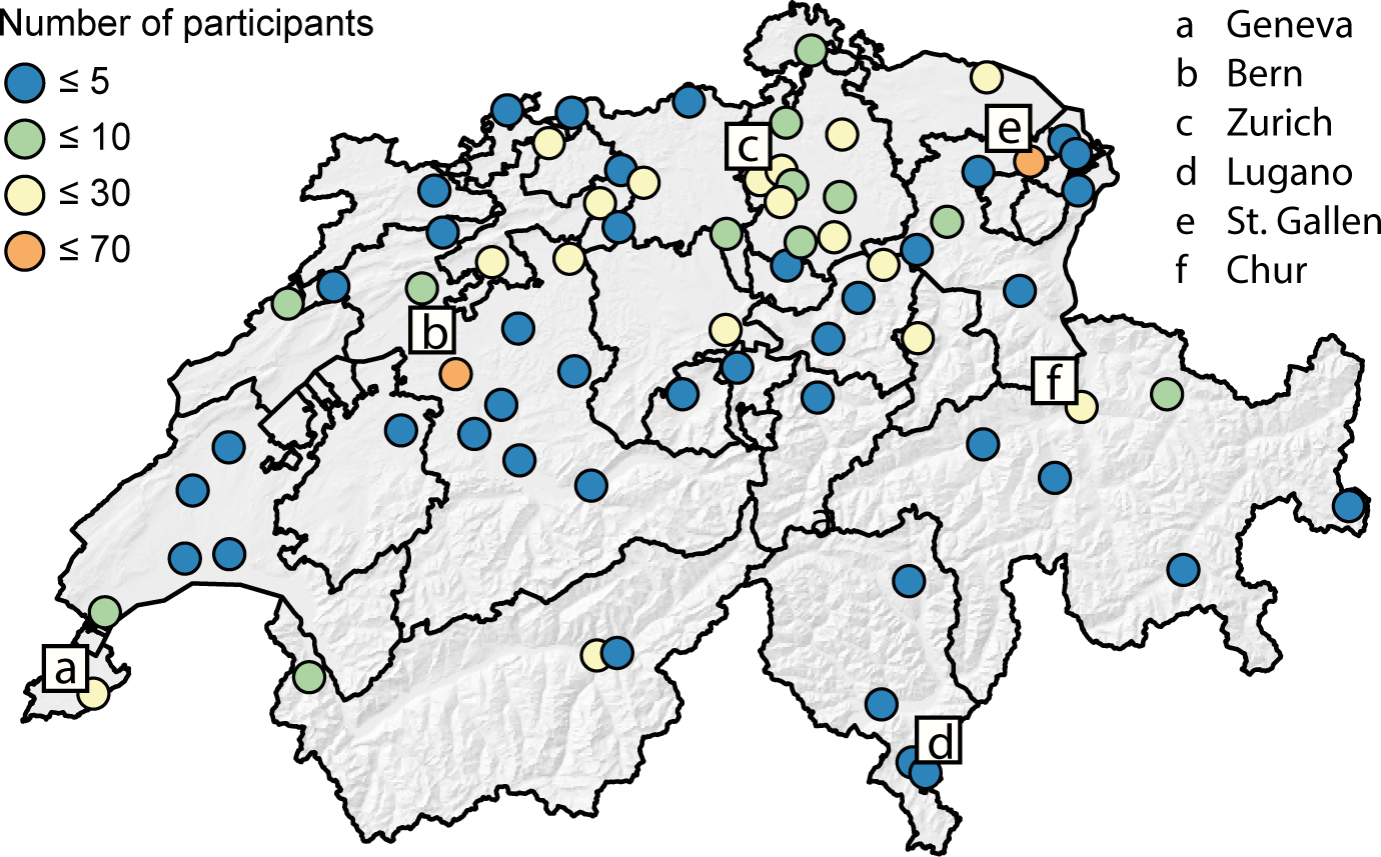
Number of physicians responding to the online questionnaire. 560 physicians from 71 Swiss hospitals working in the field of internal medicine participated in this study. The illustration indicates the number of participating physicians of the respective hospital.

71 hospitals (of 113, 64%) participated in the study with a total of 572 physicians. Twelve questionnaires were excluded from the analysis, of which 5 only logged in, but didn’t fill out the questionnaires, 4 were duplicates and 3 contained repeated apparent misstatements. Finally, a total of 560 physicians working in the field of internal medicine [28] were included in the final statistical analyses (**Fig 1**). 297 (53%) of the participants were residents, 263 (47%) attending physicians (**Table 1**). The majority of participants lived in the German-speaking part of Switzerland (n = 484, 86%). They attended their education mainly at the medical schools of Zurich (n = 163, 29%) and Berne (n = 109, 20%), or at a medical school outside of Switzerland (n = 146, 26%). Most participants (n = 480, 86%) worked in a non-university hospital.

**Table 1:** Baseline characteristics of the participants*.

	All physicians (n = 560)	Resident physicians (n = 297, 53%)	Attending physicians (n = 263, 47%)
Female sex, no. (%)	289 (51.6)	185 (62.3)	104 (39.5)
Median age, years (IQR)	34 (29 to 42)	30 (28 to 32)	42 (37 to 51)
Clinical experience, years (IQR)	6 (3 to 15)	3 (2 to 4)	15 (10 to 23)
Language region ^†^			
German, no. (%)	484 (86.4)	259 (87.2)	225 (85.6)
French, no. (%)	57 (10.2)	31 (10.4)	26 (9.9)
Italian, no. (%)	11 (2.0)	2 (0.7)	9 (3.4)
Bilingual (French/German), no. (%)	8 (1.4)	5 (1.7)	3 (1.1)

* Variables are presented as median (IQR) or mean (SD).

^†^ According to the Swiss Federal Office for Statistics.

### Diagnosis of anemia

**Fig 2:**
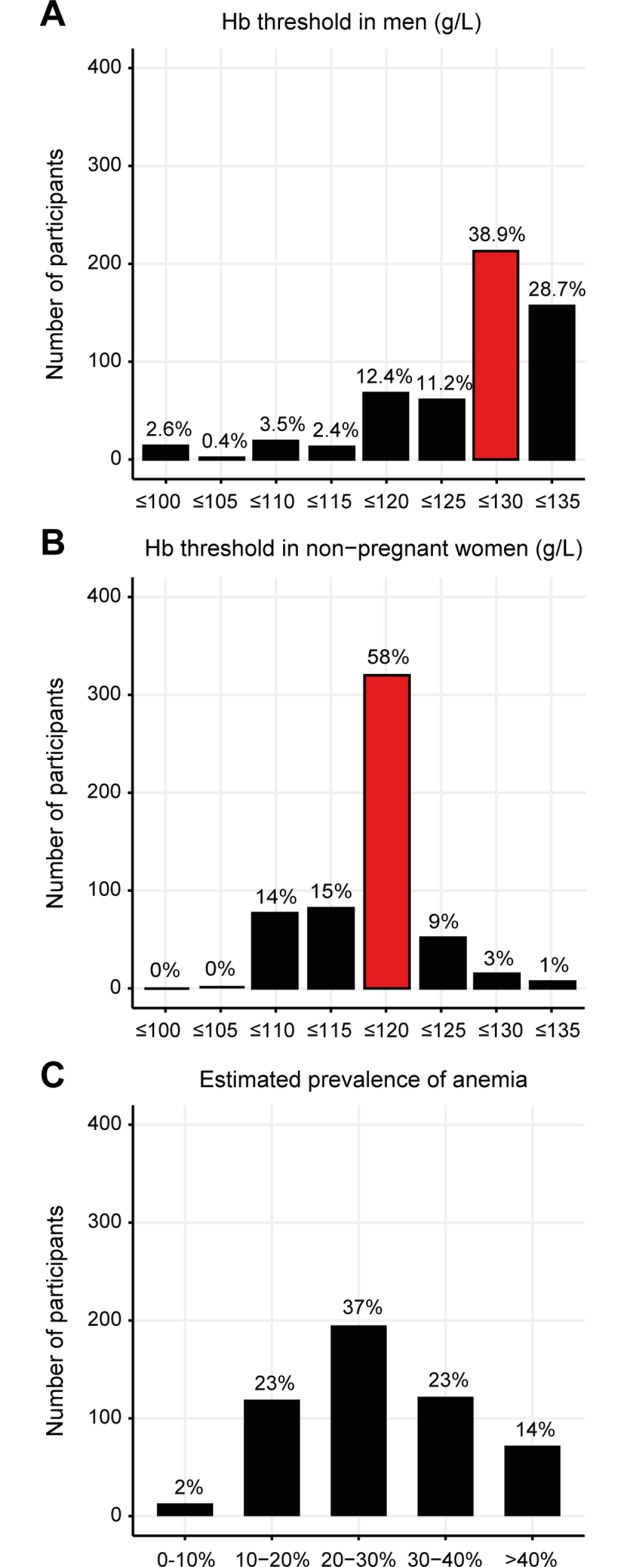
Definition of anemia. Threshold of hemoglobin level for men (**A**) and non-pregnant women (**B**), below which the presence of an anemia was diagnosed. The red bar indicates the threshold according to the definition of the World Health Organization. Estimated prevalence of anemia in the physician’s own patient population (**C**).

Anemia in men (hemoglobin concentration of ≤130 g/L) was correctly stated in 39% (213 of 547 participants), and in non-pregnant women (hemoglobin concentration of ≤120 g/L in 58% (320 of 554). Lower hemoglobin thresholds than the above mentioned were indicated by 32% in men (n = 177) and 29% in non-pregnant women (n = 159) (**Fig 2**). Physicians who studied outside of Switzerland defined anemia at a lower hemoglobin level than their Swiss colleagues (p = 0.036 for the level of male patients, **S1 Table**; p = 0.021 for the level of female patients, **S2 Table**). Eighty-nine percent (n = 462) indicated that they routinely screen for anemia on hospital admission. Eleven percent (n = 57) only screen for anemia before interventional procedures or in case of symptoms suggesting the presence of anemia. Most physicians (37%) estimated the prevalence of anemia in their patients at 20–30% (**Fig 2**).

### Guidelines on administration of red blood cells

**Fig 3:**
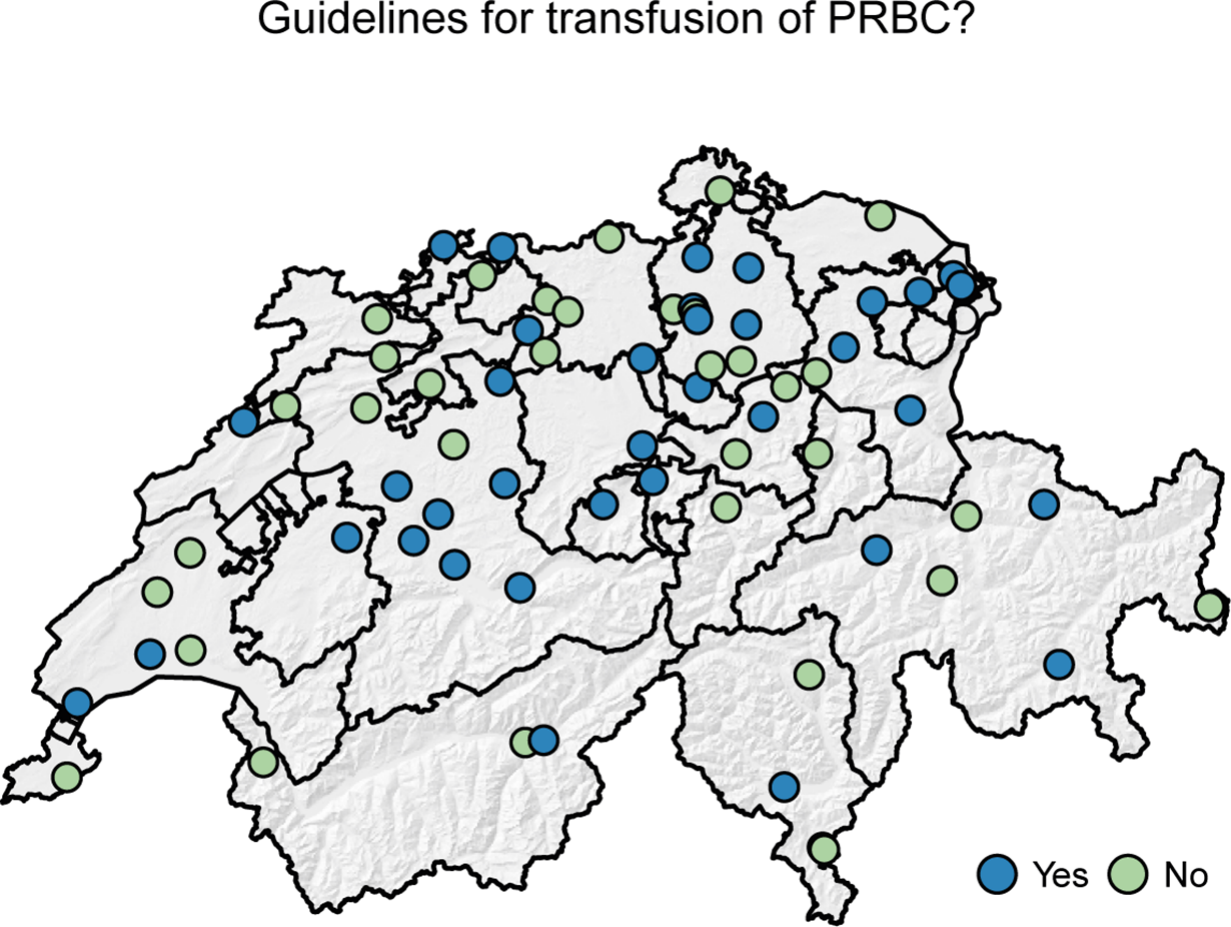
Availability of guidelines for transfusion of packed red blood cells in the physicians’ work place. Regional differences are illustrated using coloured dots which represent teaching hospitals participating in the questionnaire.

Half of the physicians (50%, n = 265) reported that local transfusion guidelines for RBC administration were not available. Moreover, there were significant differences in the availability of local transfusion guidelines among the different cantonal areas in Switzerland (**Fig 3**, p<0.001). Since there were no Swiss guidelines available, we provided the study participants with a selection of internationally accepted transfusion guidelines [8, 15, 29–31]. The participants had to indicate, which of these guidelines were known and which had an impact on their decisions to transfuse RBC. Seventeen percent reported (n = 95) to be guided by the recommendations of Carson et al. [29] Other recommendations by Klein et al. [8], Liumbruno et al. [15], Murphy et al. [30] or the German Medical Association [31] were each mentioned by less than 5% (n = 17 to 25) of all physicians. More than 69% of the interviewed physicians (n = 388) indicated that they did not know or use any of the above-mentioned guidelines to administer RBC. The majority of the physicians (n = 433, 84%) participating in our online questionnaire supported the development of national clinical practice guidelines for RBC transfusions.

### Self-perception of red blood cell (RBC) transfusion practice in anemia

**Fig 4:**
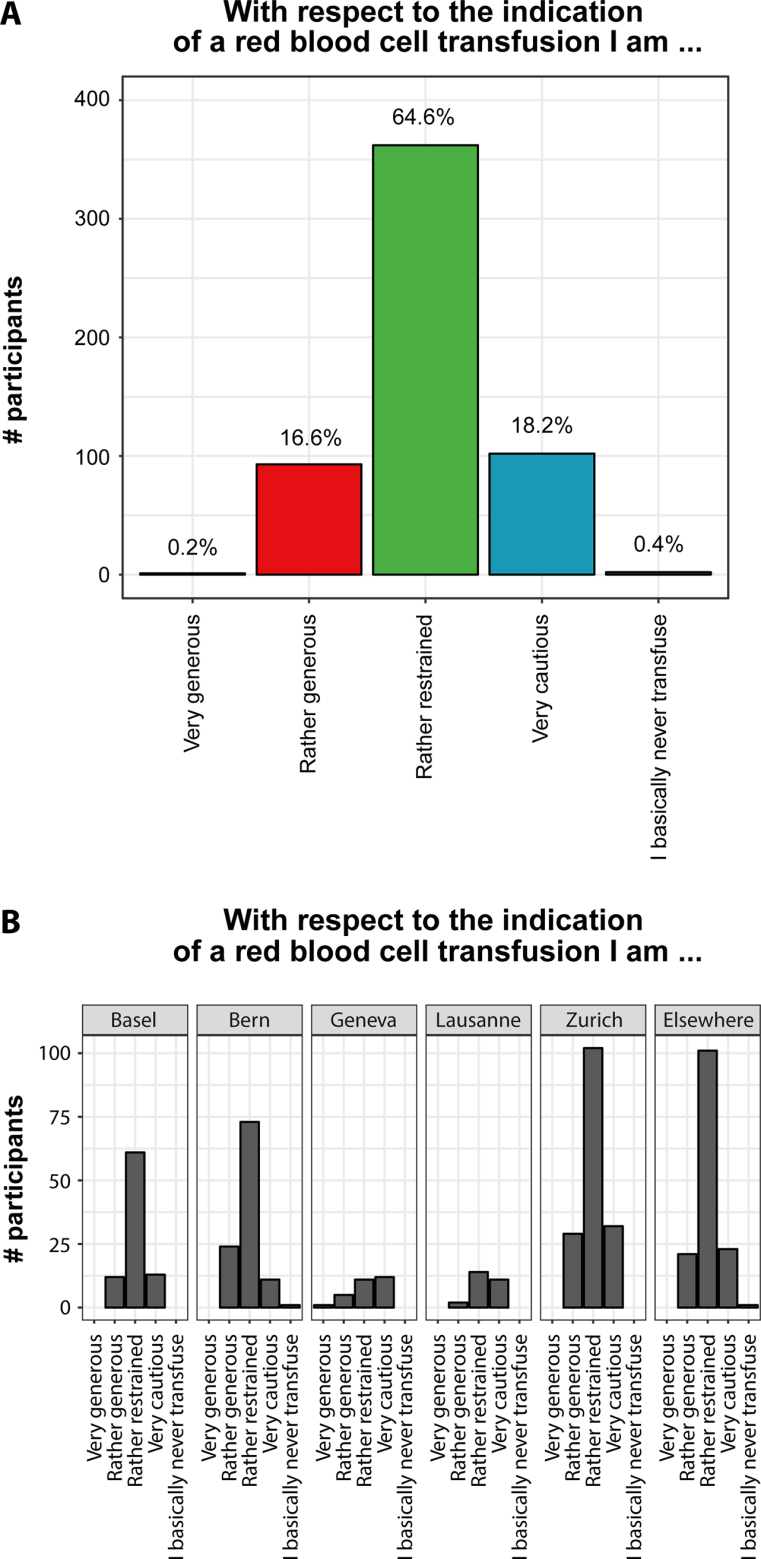
Self-perception of red blood cell (RBC) transfusion practice in anemia. Self-perception of red blood cell (RBC) transfusion practice in anemia (**A**). Association between the physician’s self-perception and the place of study (**B**).

**Fig 5:**
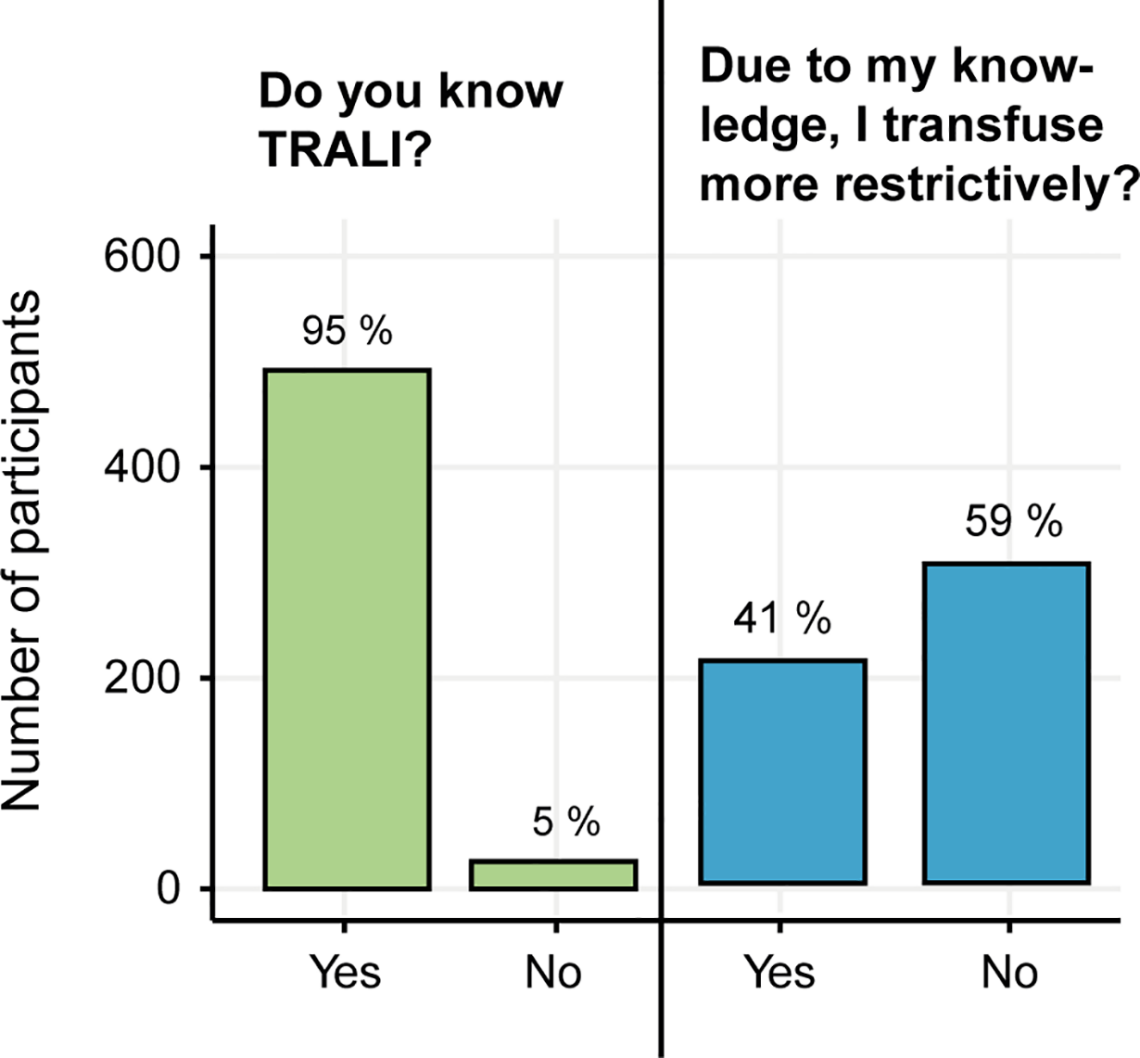
Knowledge on transfusion related acute lung injury (TRALI). Physicians knowledge of TRALI and if their knowledge on TRALI influences their transfusion practice.

Most participants (83%, n = 466) deemed themselves as restrictive when transfusing RBC. However, 17% (n = 94) reported to transfuse rather liberally to very liberally (**Fig 4**). Male doctors and doctors who studied in the French part of Switzerland (Lausanne and Geneva) described themselves to be more restrictive in transfusing RBC (p-values: 0.007 and 0.024, see **S3 Table**). Neither the years of clinical experience, nor being a resident or attending physician had an influence on the self-perceived transfusion behavior (p = 0.615 and p = 0.053). Neither did doctors working in a university hospital considered themselves more or less restrictive when transfusing RBC than doctors in non-university hospitals (p = 0.131). Almost all physicians (n = 493, 95%) were familiar with the term “transfusion-related acute lung injury (TRALI) (**Fig 5**), but only 41% (n = 213) reported that their knowledge on TRALI influenced their current transfusion practice. From a predefined list, the participants were asked to select the most important transfusion triggers. Among the most commonly indicated parameters influencing the decision to administer blood were the severity and dynamic of the anemia (43%, n = 128 and 49%, n = 147 respectively) and the presence of coronary artery disease (68%, n = 203). The majority of participants (n = 401, 75%) estimated the increase of the hemoglobin value to be 10 g/L after the administration of one unit of RBC.

### Case vignette 1. Hemodynamically stable, anemic patient without cardiovascular disease

**Fig 6:**
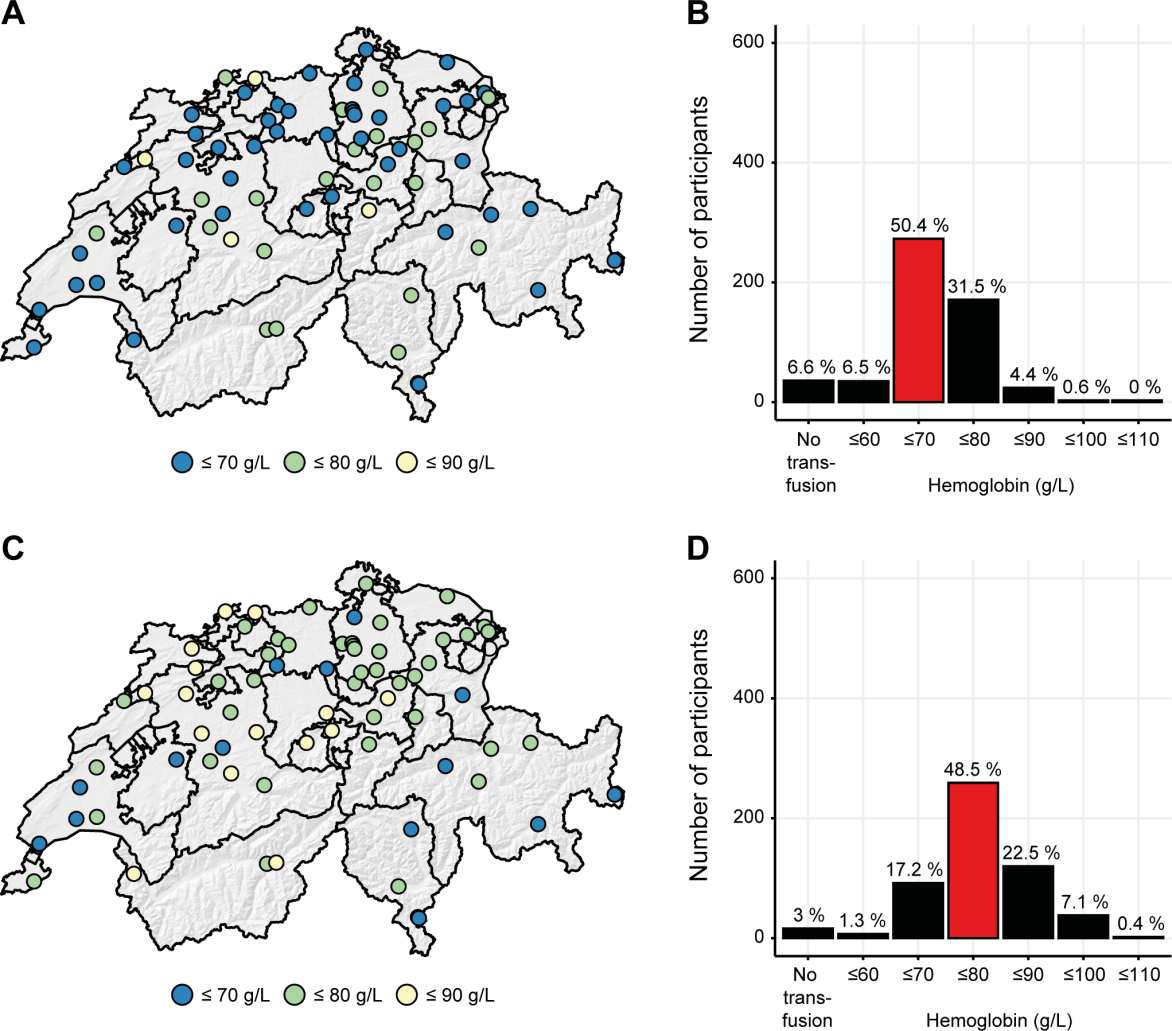
Transfusion threshold in anemia without acute bleeding and with stable oxygen delivery. Case vignette #1. In a 72-year-old male patient with stable vital parameters hospitalized due to pneumonia anemia was detected. There were no signs of active bleeding or coronary artery diseases **(A, B)**. Case vignette #2. Anemia was detected in an 84-year-old female patient with stable vital parameters suffering from osteoporotic fracture of TH12 vertebra. She had known diabetes mellitus type 2, arterial hypertension, and stable coronary artery disease **(C, D)**. The red bar indicates the recommendation of the AABB guidelines. Regional differences are illustrated on the maps using colored dots which represent teaching hospitals participating in the questionnaire.

In the first vignette, anemia was detected in a 72-year-old male patient with stable vital parameters hospitalized due to pneumonia. There were no signs of active bleeding and no history of coronary artery disease. A majority of physicians (n = 273, 50.4%) indicated a hemoglobin level of ≤ 70 g/L as the transfusion threshold in this clinical situation, while 31.5% (n = 171) reported a threshold of ≤ 80 g/L (**Fig 6**). Attending physicians indicated that they would transfuse at a lower hemoglobin threshold compared to residents (p = 0.029). The place of study significantly influenced the decision to administer RBC: physicians who studied in Basel, Berne or outside of Switzerland were much more liberal in transfusing RBC compared to physicians who studied in Zurich. Furthermore, significant regional differences were found (p = 0.010). The majority of physicians in the regions of Berne, Valais and most cantons of the central part of Switzerland set the transfusion threshold at a hemoglobin concentration of ≤ 80 g/L in this clinical situation, while in the other parts of Switzerland a threshold at a concentration of ≤ 70 g/L was reported.

### Case vignette 2. Hemodynamically stable, anemic patient with stable, pre-existing cardiovascular disease

In the second case vignette, anemia was detected in an 84-year-old female patient with stable vital parameters suffering from an osteoporotic fracture of the 12^th^ thoracic vertebra. She had a known diabetes mellitus type 2, arterial hypertension and stable coronary artery disease. In this case vignette, most of the clinicians (n = 259, 48.5%) reported to set the transfusion threshold at a hemoglobin level ≤ 80 g/L. 22.5% (n = 120) indicated a transfusion threshold ≤ 90 g/L hemoglobin, while 17.2% (n = 92) would transfuse below a hemoglobin level ≤ 70 g/L (**Fig 6**). In accordance to case vignette 1, regional differences between the region of Berne, Valais and the western part of central Switzerland were observed compared to the other regions of Switzerland (p = 0.007). The majority of physicians in these regions would administer RBC at a hemoglobin concentration of ≤ 90 g/L in this clinical situation, while the other parts of Switzerland stated a transfusion threshold at a concentration of ≤ 80 g/L.

### Case vignette 3. Hemodynamically stable, anemic patient with acute coronary syndrome

**Fig 7:**
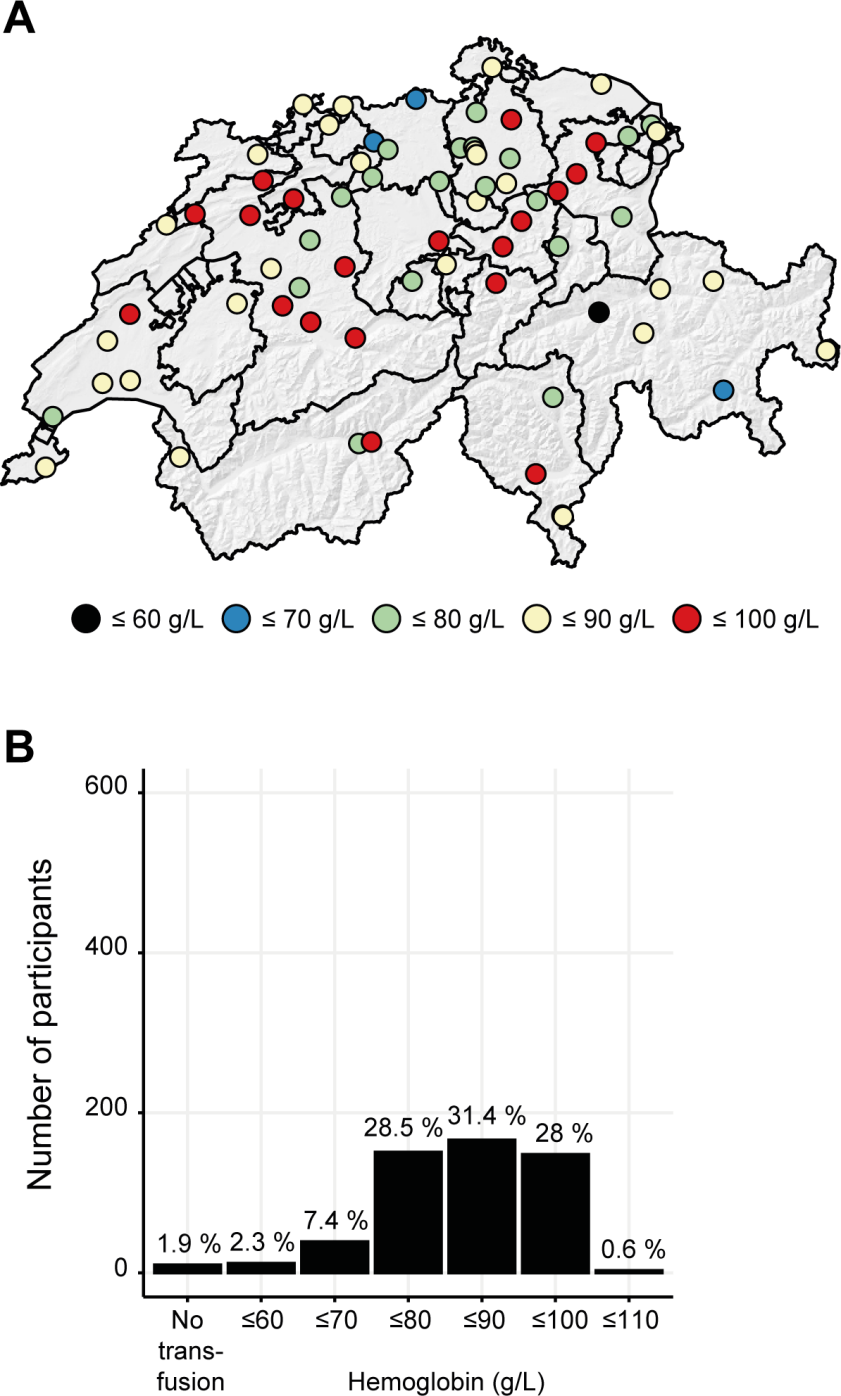
Transfusion threshold in anemic patients suffering from an acute coronary syndrome. Case vignette #3. A 64-year-old patient with stable vital parameters and without the presence of an active bleeding is hospitalized due to acute coronary syndrome. **(A, B)**. Regional differences are illustrated on the maps using colored dots which represent teaching hospitals participating in the questionnaire.

In the third vignette, anemia was diagnosed in a 64-year-old male patient with stable vital parameters hospitalized due to an acute coronary syndrome (**Fig 7**). In this case vignette, 31.4% (n = 166) of the physicians would transfuse at a hemoglobin concentration of ≤ 90 g/L, while 28.5% (n = 151) indicated a transfusion threshold of ≤ 80 g/L and 28% (n = 148) a threshold ≤ 100 g/L. Again, a regional difference between eastern cantons and the rest of Switzerland was observed (p = 0.005). Most clinicians in the cantonal area of St. Gallen, Grisons, and Glarus would transfuse RBC at a hemoglobin concentration of ≤ 80 g/L, while most other cantons indicated a transfusion threshold of ≤ 90 g/L.

## Discussion

In this cross-sectional study, we performed a nation-wide assessment on how general internal medicine physicians in teaching hospitals of Switzerland define anemia and which factors influence the decision to transfuse RBC in non-acutely bleeding patients. Furthermore, general knowledge and implementation of transfusion guidelines were assessed. Among the 560 participating physicians of 71 hospitals, anemia was defined at very diverging hemoglobin values; approximately 50% of participants did not define anemia according to the World Health Organization. Approximately 50% of participants reported not to transfuse according to international guidelines. The decision to transfuse was influenced by both, clinical and non-clinical factors, such as place of study and regional area of the hospital. Approximately 50% of the participants stated that there were no local transfusion guidelines for RBC administration. The development of national clinical practice guidelines for RBC transfusion was supported by the vast majority of physicians.

### Definition of anemia

Anemia was defined at very diverging hemoglobin values, which in about 50% did not match the WHO definition of anemia (hemoglobin level of below 120 g/L in non-pregnant women and below 130 g/L in men). Physicians who studied outside of Switzerland defined the threshold for anemia in men and in non-pregnant women at a lower hemoglobin level than their Swiss counterparts. This is not surprising as a manifold of definitions regarding the lower limit of normal blood hemoglobin concentration exist in the literature [32]. There is still an ongoing debate and varying acceptance of the WHO criteria of anemia. Instead, age-, race- and even altitude-adjusted definitions of anemia have been suggested [33, 34]. Furthermore electronic medical record (EMR) interfaces mark laboratory tests as abnormal nowadays, therefore normal values don’t have to be known any more.

### Transfusion guidelines

A plethora of clinical practice guidelines related to blood transfusions exists [12, 14, 29–31, 35–40]. This may have led to the fact that transfusion practice was handled very heterogeneously. In our study, most of the interviewed physicians indicated that they use none of the internationally most debated guidelines to administer RBC. However, most guidelines agree that RBC transfusion is clearly indicated at hemoglobin levels below 60 g/L and usually indicated at levels below 70–80 g/L (restrictive transfusion strategy). Furthermore, almost all guidelines agree that RBC are rarely or not indicated at hemoglobin levels above110 g/L [12]. In view of our results, there is certainly potential for reduction in the amount of RBC transfusions performed.

Politsmakher et al. showed that the implementation of a hospital monitoring program reduced the rate of RBC transfusion by 38.1% in his study setting, which was associated with a decrease in morbidity and mortality [41, 42]. Similar to our study but in the intensive care setting Hebert et al. conducted a survey among physicians using case scenarios which showed, that 85% of physicians had modified their approach to transfusion, primarily in response to the publication of a major Canadian clinical trial and institutional guidelines [43]. 50% of the physicians reported the absence of local transfusion guidelines for RBC administration. At present, no Swiss national guidelines exist regarding RBC transfusion practice in patients without acute blood loss [42]. The majority of the interviewed physicians (84%) would support the development of national treatment recommendations on transfusion of RBC in non-acutely bleeding patients with anemia. It is unclear whether the publication of national guidelines ultimately translate into different transfusion practice. However, there is evidence from Canadian physicians changing their transfusion practice primarily in response to the publication of national trials and institutional guidelines [43], which is why we advocate the creation of either national transfusion guidelines or the endorsement of published, evidence-based guidelines in countries that do not have a transfusion guideline from national societies or organizations, or both.

### Transfusion of RBC

Due to confounding by indication, it is difficult to establish a link between morbidity, mortality and transfusion of RBC solely based on observational data. Taking this into account, there is some limited evidence of a reduction of morbidity, mortality and costs associated with a more restrictive transfusion practice in stable medical patients [44]. In our study, almost every fifth participating physician reported to transfuse rather liberal to very liberal. The place of study, as well as the regional area of the hospital highly influenced how participants indicated their decisions to transfuse. Regional differences in medical practice between the different parts of Switzerland have previously been shown with regard to antibiotic use [45]. Switzerland consists of four different national language regions, which might influence the way medicine is practiced. Differences in transfusion practice might also arise from potential differences in training between the five university centers of Switzerland, but also by the fact that many physicians trained outside of Switzerland (in Austria, France, Germany, or Italy) and are working in Switzerland in accordance with the freedom of movement agreement with the European union. There is no general practice of reviewing appropriateness of transfusions by Swiss hospitals, which might explain the higher median transfusion rate in Switzerland compared to other European countries [46]. Attending physicians, male doctors and physicians who studied in the French part of Switzerland thought themselves to be more restrictive, but in fact, they did not choose lower hemoglobin thresholds in the clinical vignettes in comparison to their counterparts. The majority of participants indicated that in clinical practice the decision to transfuse was mainly influenced by the severity and dynamic of anemia (hemoglobin level) and by the presence of a coronary artery disease. This is in line with the internationally most accepted recommendations which in particular mention the hemoglobin level, ST-segment alterations and wall motion abnormalities as important transfusion triggers [47]. Especially, in patient populations at risk for an ischemic event, restrictive transfusion strategies have to be applied with caution and might not be beneficial for the patient’s outcome [48]. Transfusion-related acute lung injury (TRALI) is the most common cause of morbidity and mortality associated with transfusion of blood products [49]. We were astonished by the fact that most doctors were aware of TRALI, but only 40% indicated that their transfusion practice was influenced by it. The incidence of TRALI associated to RBC transfusion is relatively low compared to the incidence associated with transfusion of plasma-rich products [50], which might be an explanation for clinicians not fearing TRALI as much as the literature might suggest. Leukoreduction might be one of the reasons having helped to decrease some of the harmful effects of blood transfusion [51], but the quality of blood transfusions, however, is very dependent on the blood service system a country or geographical local has in place. Physicians’ knowledge on the basics of transfusion physiology of RBC leaves room for improvement: One in five physicians did not know what rise in hemoglobin level should be expected after RBC administration. The maximum shelf life of RBC, which is up to 42 days, is not known by 78% of the study participants [52]. The underlying causes for the lack of knowledge and awareness with regard to blood transfusions have to be investigated in further research. Altogether, our findings suggest that the educational aspects with regard to blood transfusion should be better addressed in Switzerland.

### Case vignettes

We compared our case vignettes to the AABB guidelines (formerly, American Association of Blood Banks) [53]. In both cases of a hospitalized, hemodynamically stable patient without cardiovascular disease (case vignette 1, transfusion threshold < 70 g/L) and a patient with stable, pre-existing cardiovascular disease (case vignette 2, transfusion threshold < 80 g/L), almost 50% of the physicians would transfuse according to the AABB guidelines. A large proportion of physicians would however transfuse too liberally at higher hemoglobin levels in this vignette. In the third clinical vignette, covering an anemic patient with acute coronary heart disease, no recommendation is given by the AABB guidelines due to limited available evidence. This is reflected in the answers given by the participating physicians. Similar proportions would transfuse at hemoglobin levels of < 80 g/L, < 90 g/L and < 100 g/L with no clear preference. It remains unclear how concerns to cause Transfusion Associated Circulatory Overload influenced the participant’s responses to the third case vignette [54]. In general, less may be better when it comes to blood transfusion. However, in any decision to transfuse, one must weigh the risks and benefits associated with transfusion against, those associated with anemia. Transfusion decisions need not only to follow the transfusion threshold, but to consider individual patient characteristics, including age and presence of coronary artery disease, to estimate a benefit from transfusion.

### Strengths and limitations

The present investigation gives, for the first time, insights on how hospital physicians think they diagnose and treat anemia in non-bleeding, hospitalized patients. Each clinical director (general internal medicine of teaching hospitals) was asked to distribute our information letter to all physicians of his or her department, containing the link to the online questionnaire. We can therefore only estimate how many physicians have actually received an invitation to participate in the questionnaire. Unfortunately, due to the structure of the Swiss Medical Association (FMH) registry [18] it is not possible to adequately determine the proportion of participating physicians, as the register does not distinguish between acute care and rehabilitation hospitals and does not clearly depict the current number of employed physicians per annum. However, 560 physicians from 71 hospitals (64% of all hospitals with an internal medicine ward) responded to the questionnaire, which corresponds to a response rate above the average for nation-wide surveys [55]. Average response rate for survey studies that utilized data collected from organizations has been reported among 36 percent [56]. The geographical distribution of the physicians who replied to the questionnaire demonstrates that not only large centers (such as Zurich, Berne or Geneva), but also smaller hospitals are well represented within our survey. It is noteworthy that most of the attending physicians in Switzerland underwent an additional specialty training (e.g. endocrinology, gastroenterology, or cardiology) in addition to their training in general internal medicine. This secondary specialty training might have significantly influenced their responses the questionnaire items. The geographical distribution (**Fig 1**) shows that all regions of Switzerland are well represented in the survey, which is a strength of this study. However, based on our analysis, we have to assume that especially the university hospitals (Lausanne, Basel) and some parts of western Switzerland might have been underrepresented in our study. We accounted in our analysis for potential differences between university versus non-university hospitals. The analysis, however, was not stratified for the number of physicians per hospital. Furthermore, the results of our study represent the individual doctor’s theoretical self-assessment of their personal transfusion practice in non-bleeding patients, which might very much differ from their actual transfusion practice. This is partially reflected in the above-mentioned divergence of some doctors self-reported restrictive transfusion behavior, which was not reflected in their transfusion practice in the clinical vignettes. On the other hand, the vignettes, represent theoretical cases and some information e.g. on the interval to develop anemia and expectance of Hb recovery upon administration nutrients if indicated (iron, vitamin B12, folinic acid) has not been included to reduce complexity. The present article describes the result from a cross-sectional survey and therefore only provides a snapshot of reported transfusion practices. In addition, the physicians’ answers might only partially reflect effective decisions in clinical practice.

## Conclusion

This study highlights the lack of adherence to current transfusion guidelines in Switzerland. Identifying and subsequently correcting this deficit in knowledge translation may have a significant impact on patient care.

## Supporting information

S1 Appendix Static version of the questionnaire. (DOCX)

S1 Table Definition of anaemia in non-bleeding, hospitalized male patients. (DOCX)

S2 Table Definition of anaemia in non-pregnant, non-bleeding, hospitalized female patients. (DOCX)

S3 Table Cumulative link mixed model analysis on the attitude to transfuse packed red blood cells. (DOCX)

S4 Table Cumulative link mixed model analysis with regard to the haemoglobin threshold to transfuse in case vignette 1. (DOCX)

S5 Table Cumulative link mixed model analysis with regard to the haemoglobin threshold to transfuse in case vignette 2. (DOCX)

S6 Table Cumulative link mixed model analysis with regard to the haemoglobin threshold to transfuse in case vignette 3. (DOCX)

S1 Dataset Raw dataset. (XLSX)
