# Supplementary material for: Transfusion practice in anemic, non-bleeding patients: Cross-sectional survey of physicians working in general internal medicine teaching hospitals in Switzerland
Source: PLoS One. 2018 Jan 30;13(1):e0191752. doi: 10.1371/journal.pone.0191752 (PMC5790246; doi:10.1371/journal.pone.0191752)
Supplement: S1 Appendix — (DOCX) [file pone.0191752.s001.docx]

**S1 Appendix**

***Static version of the questionnaire***

1. With respect to the indication of a red blood cell transfusion I am ...

- Very generous
- Rather generous
- Rather restrained
- Very cautious
- I basically never transfuse

1. Below which haemoglobin threshold, do you diagnose an anaemia in non-pregnant women?

- ≤ 13.5 g/dL
- ≤ 13.0 g/dL
- ≤ 12.5 g/dL
- ≤ 12.0 g/dL
- ≤ 11.5 g/dL
- ≤ 11.0 g/dL
- ≤ 10.5 g/dL
- ≤ 10.0 g/dL

1. Below which haemoglobin threshold, do you diagnose an anaemia in men?

- ≤ 13.5 g/dL
- ≤ 13.0 g/dL
- ≤ 12.5 g/dL
- ≤ 12.0 g/dL
- ≤ 11.5 g/dL
- ≤ 11.0 g/dL
- ≤ 10.5 g/dL
- ≤ 10.0 g/dL

1. In normal-weight adults without active blood loss, which rise in the haemoglobin concentration is to be expected in 2-24 h after the transfusion of an erythrocyte concentrate?

- 0.5 g/dL
- 1.0 g/dL
- 1.5 g/dL
- 2.0 g/dL
- 3.0 g/dL
- Don’t know

1. In normal-weight adults without active blood loss, which rise in the haematocrit level is to be expected in 2-24 h after the transfusion of an erythrocyte concentrate?

- 1%
- 2%
- 3%
- 4%
- 5%
- 10%
- Don’t know

1. Does your clinic provide guidelines and / or in-house protocols for the transfusion of red blood cells in anaemic patients?

- Yes
- No

1. How high do you estimate the prevalence of anaemia in your patients?

- 0-10%
- 10-20%
- 20-30%
- 30-40%
- >40%

1. With respect to the transfusion of red blood cells, I am guided by the following guidelines / recommendations? (multiple answers are possible)

- Carson JL et al. Red Blood Cell Transfusion: A Clinical Practice Guideline from the AABB. Ann Intern Med. 2012 Jul 3; 157 (1): 49-58.
- Liumbruno G et al. Recommendations for the transfusion of red blood cells. Blood Transfus. 2009 January; 7(1): 49–64.
- Klein HG, Spahn et al. Red blood cell transfusion in clinical practice. Lancet 2007; 370: 415–26.
- Querschnitts-Leitlinien (BÄK) zur Therapie mit Blutkomponenten und Plasmaderivaten. 4. Auflage 2008. Bundesärztekammer, Deutschland.
- Murphy MF et al. British Committee for Standards in Haematology, Blood Transfusion Task Force. Guidelines for the clinical use of red cell transfusions. Br J Haematol. 2001;113:24-31.
- None of the above guidelines

1. Do you know TRALI (transfusion-related acute lung injury)?

- Yes
- No

1. Do you transfuse more restrictively due to your knowledge about TRALI (transfusion-related acute lung injury)?

- Yes
- No

1. Which parameters do you consider when deciding to transfuse red blood cells in an anaemic patient without the presence of an active bleeding? (Please select three main parameters)

- Haemoglobin / severity of anaemia
- Coronary artery disease
- Dynamics of anaemic state
- Cause of anaemia
- Dyspnoea
- Patient’s age
- Cardiac function
- Heart rate
- ECG alterations
- Clinical signs of volume depletion

1. Do you support the development of national treatment recommendations on transfusion of red blood cells concentrates non-actively bleeding patients having an anaemia?

- Yes, national recommendations would be necessary
- No, national recommendations are not necessary

**Case Vignette 1:**

In a 72-year, hemodynamically stable patient with pneumonia, an anaemia has been detected. There are no signs of an active bleeding, nor is a coronary heart disease known. Below which haemoglobin threshold do you decide to transfuse packed red blood cells?

- ≤ 11.0 g/dL
- ≤ 10.0 g/dL
- ≤ 9.0 g/dL
- ≤ 8.0 g/dL
- ≤ 7.0 g/dL
- ≤ 6.0 g/dL
- No transfusion

**Case Vignette 2:**

In an 84-year old, hemodynamically stable patient with an osteoporotic fracture of Th12, an anaemia has been detected. According to his personal history, he suffers from arterial hypertension and coronary heart disease and diabetes mellitus. There are no signs of an active bleeding or for the presence of an acute coronary syndrome. Below which haemoglobin threshold do you decide to transfuse packed red blood cells?

- ≤ 11.0 g/dL
- ≤ 10.0 g/dL
- ≤ 9.0 g/dL
- ≤ 8.0 g/dL
- ≤ 7.0 g/dL
- ≤ 6.0 g/dL
- No transfusion

**Case Vignette 3:**

A 64-year-old, hemodynamically stable patient is hospitalized due to an acute coronary syndrome. Below which haemoglobin threshold do you decide to transfuse packed red blood cells?

- ≤ 11.0 g/dL
- ≤ 10.0 g/dL
- ≤ 9.0 g/dL
- ≤ 8.0 g/dL
- ≤ 7.0 g/dL
- ≤ 6.0 g/dL
- No transfusion
