# Supplementary material for: Transfusion practice in anemic, non-bleeding patients: Cross-sectional survey of physicians working in general internal medicine teaching hospitals in Switzerland
Source: PLoS One. 2018 Jan 30;13(1):e0191752. doi: 10.1371/journal.pone.0191752 (PMC5790246; doi:10.1371/journal.pone.0191752)
Supplement: S1 Table — (DOCX) [file pone.0191752.s002.docx]

**S1 Table.** Definition of anaemia in non-bleeding, hospitalized male patients

|  | Odds ratios (95% confidence interval) | Pr(>\|z\|) |
| --- | --- | --- |
| *Fixed effects* |  |  |
| Clinical experience, years | 1.00 (0.98 to 1.01) | 0.778 |
| Attending physician | 1.06 (0.77 to 1.46) | 0.761 |
| Male sex | 0.97 (0.77 to 1.21) | 0.801 |
| Working in a non-university hospital | 1.12 (0.79 to 1.59) | 0.595 |
| Place of study |  |  |
| Basel | 0.92 (0.65 to 1.30) | 0.681 |
| Berne | 1.02 (0.74 to 1.42) | 0.908 |
| Geneva | 1.01 (0.57 to 1.78) | 0.978 |
| Lausanne | 1.29 (0.74 to 2.22) | 0.454 |
| Outside of Switzerland | 0.65 (0.49 to 0.88) | 0.021 * |
| *Random effects* |  |  |
| Variance by cantonal area (SD) | 2.5 (1.6) | 1.0 |

The table shows estimates and corresponding 95% confidence intervals. Female residents who studied in Zurich and are now working in a university hospital have been defined as the control group in the mixed model. Dependent variable: threshold in haemoglobin levels to diagnose the presence of anaemia. AIC: 2084.317; n=560; * p < 0.05
