# Supplementary material for: Transfusion practice in anemic, non-bleeding patients: Cross-sectional survey of physicians working in general internal medicine teaching hospitals in Switzerland
Source: PLoS One. 2018 Jan 30;13(1):e0191752. doi: 10.1371/journal.pone.0191752 (PMC5790246; doi:10.1371/journal.pone.0191752)
Supplement: S2 Table — (DOCX) [file pone.0191752.s003.docx]

**S2 Table.** Definition of anaemia in non-pregnant, non-bleeding, hospitalized female patients

|  | Odds ratios (95% confidence interval) | Pr(>\|z\|) |
| --- | --- | --- |
| *Fixed effects* |  |  |
| Clinical experience, years | 1.00 (0.98 to 1.03) | 0.808 |
| Attending physician | 0.81 (0.55 to 1.21) | 0.392 |
| Male sex | 1.33 (1.00 to 1.77) | 0.099 |
| Working in a non-university hospital | 1.53 (1.00 to 2.33) | 0.098 |
| Place of study |  |  |
| Basel | 0.84 (0.55 to 1.29) | 0.500 |
| Berne | 1.07 (0.71 to 1.60) | 0.789 |
| Geneva | 1.46 (0.72 to 2.99) | 0.387 |
| Lausanne | 0.97 (0.50 to 1.91) | 0.947 |
| Outside of Switzerland | 0.62 (0.43 to 0.90) | 0.036 * |
| *Random effects* |  |  |
| Variance by cantonal area (SD) | 0 (0) | 1.0 |

The table shows estimates and corresponding 95% confidence intervals. Female residents who studied in Zurich and are now working in a university hospital have been defined as the control group in the mixed model. Dependent variable: threshold in haemoglobin levels to diagnose the presence of anaemia. AIC: 1398.159; n=560; * p < 0.05
