# Supplementary material for: Transfusion practice in anemic, non-bleeding patients: Cross-sectional survey of physicians working in general internal medicine teaching hospitals in Switzerland
Source: PLoS One. 2018 Jan 30;13(1):e0191752. doi: 10.1371/journal.pone.0191752 (PMC5790246; doi:10.1371/journal.pone.0191752)
Supplement: S3 Table — (DOCX) [file pone.0191752.s004.docx]

**S3 Table.** Cumulative link mixed model analysis on the self-perception to transfuse packed red blood cells.

|  | Odds ratios (95% confidence interval) | Pr(>\|z\|) |
| --- | --- | --- |
| *Fixed effects* |  |  |
| Clinical experience, years | 1.01 (0.98 to 1.03) | 0.615 |
| Attending physician | 0.61 (0.40 to 0.93) | 0.053 |
| Male sex | 0.70 (0.52 to 0.94) | 0.048 * |
| Working in a non-university hospital | 0.63 (0.38 to 1.04) | 0.131 |
| Place of study |  |  |
| Basel | 0.97 (0.60 to 1.55) | 0.909 |
| Berne | 1.31 (0.82 to 2.11) | 0.342 |
| Geneva | 0.31 (0.13 to 0.73) | 0.024 * |
| Lausanne | 0.28 (0.13 to 0.60) | 0.007 * |
| Outside of Switzerland | 0.90 (0.60 to 1.35) | 0.666 |
| *Random effects* |  |  |
| Variance by cantonal area (SD) | 0.1 (0.3) | 0.213 |

The table shows estimates and corresponding 95% confidence intervals. Female residents who studied in Zurich and are now working in a university hospital have been defined as the control group in the mixed model. Dependent variable: Self-perception to transfuse more liberally. AIC: 991.8796; n=560; * p < 0.05
