# Supplementary material for: Transfusion practice in anemic, non-bleeding patients: Cross-sectional survey of physicians working in general internal medicine teaching hospitals in Switzerland
Source: PLoS One. 2018 Jan 30;13(1):e0191752. doi: 10.1371/journal.pone.0191752 (PMC5790246; doi:10.1371/journal.pone.0191752)
Supplement: S4 Table — (DOCX) [file pone.0191752.s005.docx]

**S4 Table.** Cumulative link mixed model analysis with regard to the haemoglobin threshold to transfuse in case vignette 1

|  | Odds ratios (95% confidence interval) | Pr(>\|z\|) |
| --- | --- | --- |
| *Fixed effects* |  |  |
| Clinical experience, years | 1.01 (0.99 to 1.04) | 0.287 |
| Attending physician | 0.59 (0.40 to 0.88) | 0.029 * |
| Male sex | 0.76 (0.57 to 1.01) | 0.115 |
| Working in a non-university hospital | 1.33 (0.78 to 2.25) | 0.381 |
| Place of study |  |  |
| Basel | 2.06 (1.30 to 3.26) | 0.009 * |
| Berne | 2.59 (1.65 to 4.07) | 0.001 * |
| Geneva | 1.24 (0.56 to 2.73) | 0.661 |
| Lausanne | 1.32 (0.58 to 2.98) | 0.579 |
| Outside of Switzerland | 2.43 (1.64 to 3.61) | < 0.001 * |
| *Random effects* |  |  |
| Variance by cantonal area (SD) | 0.2 (0.4) | 0.010 * |

The table shows estimates and corresponding 95% confidence intervals. Female residents who studied in Zurich and are now working in a university hospital have been defined as the control group in the mixed model. Dependent variable: threshold in haemoglobin levels to transfuse packed red blood cells. AIC: 1322.573; n=560; * p < 0.05
