# Supplementary material for: Transfusion practice in anemic, non-bleeding patients: Cross-sectional survey of physicians working in general internal medicine teaching hospitals in Switzerland
Source: PLoS One. 2018 Jan 30;13(1):e0191752. doi: 10.1371/journal.pone.0191752 (PMC5790246; doi:10.1371/journal.pone.0191752)
Supplement: S5 Table — (DOCX) [file pone.0191752.s006.docx]

**S5 Table.** Cumulative link mixed model analysis with regard to the haemoglobin threshold to transfuse in case vignette 2

|  | Odds ratios (95% confidence interval) | Pr(>\|z\|) |
| --- | --- | --- |
| *Fixed effects* |  |  |
| Clinical experience, years | 0.98 (0.96 to 1.00) | 0.200 |
| Attending physician | 0.86 (0.58 to 1.26) | 0.515 |
| Male sex | 0.82 (0.62 to 1.08) | 0.232 |
| Working in a non-university hospital | 0.75 (0.45 to 1.25) | 0.356 |
| Place of study |  |  |
| Basel | 1.63 (1.04 to 2.57) | 0.073 |
| Berne | 1.67 (1.08 to 2.59) | 0.053 |
| Geneva | 0.70 (0.30 to 1.62) | 0.483 |
| Lausanne | 0.96 (0.43 to 2.13) | 0.929 |
| Outside of Switzerland | 1.45 (0.99 to 2.13) | 0.108 |
| *Random effects* |  |  |
| Variance by cantonal area (SD) | 0.2 (0.4) | 0.007 * |

The table shows estimates and corresponding 95% confidence intervals. Female residents who studied in Zurich and are now working in a university hospital have been defined as the control group in the mixed model. Dependent variable: threshold in haemoglobin levels to transfuse packed red blood cells. AIC: 1442.209; n=560; * p < 0.05
