# Supplementary material for: Transfusion practice in anemic, non-bleeding patients: Cross-sectional survey of physicians working in general internal medicine teaching hospitals in Switzerland
Source: PLoS One. 2018 Jan 30;13(1):e0191752. doi: 10.1371/journal.pone.0191752 (PMC5790246; doi:10.1371/journal.pone.0191752)
Supplement: S6 Table — (DOCX) [file pone.0191752.s007.docx]

**S6 Table.** Cumulative link mixed model analysis with regard to the haemoglobin threshold to transfuse in case vignette 3

|  | Odds ratio | Pr(>\|z\|) |
| --- | --- | --- |
| *Fixed effects* |  |  |
| Clinical experience, years | 1.00 (0.98 to 1.03) | 0.792 |
| Attending physician | 1.17 (0.80 to 1.71) | 0.503 |
| Male sex | 0.88 (0.67 to 1.16) | 0.443 |
| Working in a non-university hospital | 1.16 (0.71 to 1.91) | 0.614 |
| Place of study |  |  |
| Basel | 1.27 (0.82 to 1.96) | 0.367 |
| Berne | 1.24 (0.80 to 1.91) | 0.422 |
| Geneva | 1.23 (0.57 to 2.67) | 0.653 |
| Lausanne | 1.67 (0.79 to 3.52) | 0.261 |
| Outside of Switzerland | 1.19 (0.82 to 1.71) | 0.447 |
| *Random effects* |  |  |
| Variance by cantonal area (SD) | 0.2 (0.4) | 0.005 * |

The table shows estimates and corresponding 95% confidence intervals. Female residents who studied in Zurich and are now working in a university hospital have been defined as the control group in the mixed model. Dependent variable: threshold in haemoglobin levels to transfuse packed red blood cells. AIC: 1559.808; n=560; * p < 0.05
